# The Popularisation of Medical Knowledge

**Published:** 1900-10-13

**Authors:** 


### The Hospital

A Journal of

The Medical Sciences and Hospital Administration.

Vol. XXIX.—No. 733.]

Edited by Sir Henry Burdett, K.C.B., and Solomon C. Smith, M.D., M.R.C.P.

[October 13, 1900.

### The Popularisation of Medical Knowledge.

We have long entertained a conviction that the grievances of the medical profession, which we must admit to be real, numerous, and sometimes serious, are in the main greatly due to public ignorance of the aims and intentions of medical work, of the purposes which it is calculated to fulfil, and of the benefits to the world in general which might be expected to arise from its adequate accomplishment. Prominent among the grievances is the rate of remuneration at which medical services are appraised by a large proportion of those to whom they are rendered, especially among the very numerous artisan class, and among the people who support the various so-called "provident" institutions or dispensaries, in some of which a medical man is engaged as the paid servant of the society, and is under an agreement to attend any one of its members, however well able to pay ordinary charges, by whom his assistance may be required. The popular view of the doctor and of his work has probably undergone very little change in the course of centuries. The readers of Sully's delightful "Memoirs" will remember that Henri IV. frequently suffered from attacks of illness which were manifestly the results of habits of life easily capable of being altered for the better; and that, when one of these attacks occurred, the royal physicians were sent for, not to prescribe the remedies which in their judgment were best adapted for the august patient's condition, but simply to apply those which were demanded by the public opinion of the time. They were not required to treat the king's maladies, but only to bleed him, or to purge him, or to administer an emetic, as the case might be. In other words, they were summoned to do a certain thing, as a farrier might be sent for to shoe a horse, or a carpenter to mend some broken woodwork. They were scarcely regarded in any other light than as persons possessing skill in handicraft, and therefore better able than others to do something which all could see was necessary to be done. The king would decide for himself that he ought to be purged, but would leave to the physicians the selection of the purgative and the adjustment of the dose and of the time of administration. The vulgar notion of to-day differs very little from the royal notion of the seventeenth century. A labouring man's child is ill, and the mother sends for the "club" or "dispensary" doctor. She has no conception that his ministrations constitute work of a different order from her own in darning a stocking, or from that of her husband in selecting and putting in a screw. He has only to give some "stuff" in a bottle or in a powder paper, and the capabilities of human effort will have been exhausted. If the child die, she will be free from responsibility; if it recover, she will perhaps think that it might have done that without the physic, and may even regret the very moderate expenditure which has been incurred. In any event, she will think that what the doctor has done was quite simple and easy, that she could have done it herself if she had known the name of the right "stuff" to give, and that the club or dispensary subscription was an ample recompense for the time and trouble which the doctor had bestowed. The prosperous tradesman who also subscribes to the "dispensary," or the fashionable lady who goes to Dr. Flapdoodle for his new "cure," does not, in any essential point, take a different view of the position.

It has often occurred to us that great good might be done by any competent writers who would popularise the general principles of medical science somewhat in the same way in which the principles of certain other branches of science have been popularised by their most eminent cultivators; say, for example, to select only the names of those who have passed away, by Professors Tyndall and Huxley. If this were done, the public would in time arrive at some conception, even if it were only an inadequate one, of the difficulty and complexity of many medical problems; a result from which two good issues might proceed. In the first place, some appreciation of the dishonesty of quackery, and of the dangers entailed upon its dupes, could scarcely fail to arise; and, in the second place, any convictions of this kind, although limited at first to educated people, would in time percolate to inferior strata of the community. Few better opportunities for such work will ever be afforded than those arising from the recent researches into the essential nature and the modes of diffusion of the so-called "malaria." Nothing in the physics of heat or of light is more beautiful or more interesting than the precise knowledge which has been gained of the natural history of the parasites by which this disease is caused, of their cycles of existence and multiplication in their successive hosts, and of the effects which they produce in the human organism. We cordially recommend the subject to some writer who is able to do justice to its capabilities, and who would set forth the facts and their teachings in plain English, such that it would be "understanded of the people."

